# The Pentatricopeptide Repeat Gene Family in *Salvia miltiorrhiza*: Genome-Wide Characterization and Expression Analysis

**DOI:** 10.3390/molecules23061364

**Published:** 2018-06-06

**Authors:** Heqin Li, Caili Li, Yuxing Deng, Xuwen Jiang, Shanfa Lu

**Affiliations:** 1 Institute of Medicinal Plant Development, Chinese Academy of Medical Sciences & Peking Union Medical College, No.151, Malianwa North Road, Haidian District, Beijing 100193, China; hqliaau@163.com (H.L.); licaili390@163.com (C.L.); yuxingdeng2016@163.com (Y.D.); 2 College of Agronomy, Qingdao Agricultural University, No. 700 Changcheng Road, Chengyang District, Qingdao 266109, China; jxw888@163.com

**Keywords:** *Salvia miltiorrhiza*, pentatricopeptide repeat gene, expression analysis, yeast extract and Ag^+^, salicylic acid

## Abstract

The pentatricopeptide repeat (PPR) gene family is one of the largest gene families in plants and plays important roles in posttranscriptional regulation. In this study, we combined whole genome sequencing and transcriptomes to systematically investigate *PPRs* in *Salvia miltiorrhiza*, which is a well-known material of traditional Chinese medicine and an emerging model system for medicinal plant studies. Among 562 identified *SmPPRs*, 299 belong to the P subfamily while the others belong to the PLS subfamily. The majority of *SmPPRs* have only one exon and are localized in the mitochondrion or chloroplast. As many as 546 *SmPPRs* were expressed in at least one tissue and exhibited differential expression patterns, which indicates they likely play a variety of functions in *S. miltiorrhiza*. Up to 349 *SmPPRs* were salicylic acid-responsive and 183 *SmPPRs* were yeast extract and Ag^+^-responsive, which indicates these genes might be involved in *S. miltiorrhiza* defense stresses and secondary metabolism. Furthermore, 23 salicylic acid-responsive *SmPPRs* were co-expressed with phenolic acid biosynthetic enzyme genes only while 16 yeast extract and Ag^+^-responsive *SmPPRs* were co-expressed with tanshinone biosynthetic enzyme genes only. Two *SmPPRs* were co-expressed with both phenolic acid and tanshinone biosynthetic enzyme genes. The results provide a useful platform for further investigating the roles of *PPRs* in *S. miltiorrhiza*.

## 1. Introduction

**Figure 1:**
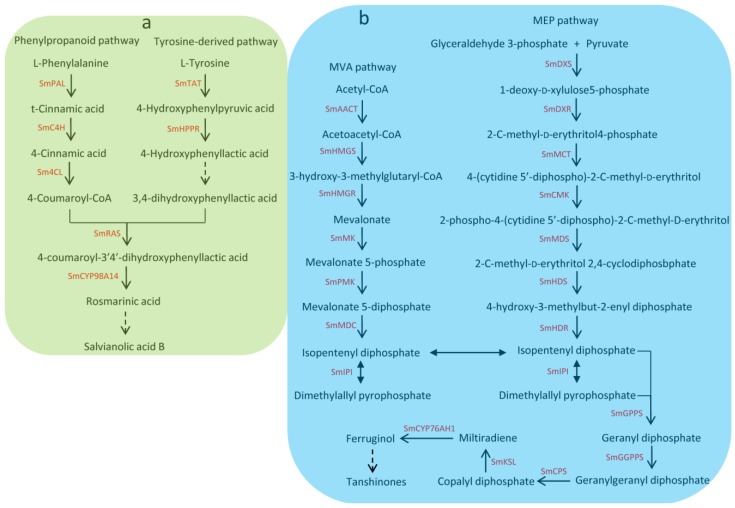
Biosynthethsis of salvianolic acids and tanshinones in *S. miltiorrhiza*. (**a**) Biosynthethsis of salvianolic acids in *S. miltiorrhiza*. SmPAL: phenylalanine ammonia-lyase, SmC4H: cinnamic acid 4-hydroxylase, Sm4CL: 4-coumarate: CoA ligase, SmTAT: tyrosine aminotransferase, SmHPPR: 4-hydroxyphenylpyruvate reductase, SmRAS: rosmarinic acid synthase; (**b**) Biosynthethsis of tanshinones in *S. miltiorrhiza*. SmAACT: acetyl-CoA C-acetyltransferase, SmHMGS: hydroxymethylglutaryl-CoA synthase, SmHMGR: hydroxymethylglutaryl-CoA reductase, SmMK: mevalonate kinase, SmPMK: 5-phosphomevalonate kinase, SmMDC: mevalonate 5-diphosphate decarboxylase, SmIPI: isopentenyl pyrophosphate isomerase, SmDXS: 1-deoxy-d -xylulose 5-phosphate synthase, SmDXR: 1-deoxy-d-xylulose5-phosphate reductoisomerase, SmMCT: 2-C-methyl-d-erythritol 4-phosphate cytidylyltransferase, SmCMK: 4-(cytidine 5’-diphospho)-2-C-methyl-d-erythritol kinase, SmMDS: 2-C-methyl-d-erythritol 2,4-cyclodiphosbphate synthase, SmHDS: 4-hydroxy-3-methylbut-2-enyl diphosphate synthase, SmHDR: 4-hydroxy-3-methylbut-2-enyl diphosphate reductase, SmGPPS: geranyl diphosphate synthase, SmGGPPS: geranylgeranyl diphosphate synthase, SmCPS: copalyl diphosphate synthase, SmKSL: kaurene synthase-like cyclase.

*Salvia miltiorrhiza* Bunge, which is a well-known traditional Chinese medicine (TCM), is widely used to treat the cardiovascular and cerebrovascular diseases [1]. With the completion of the genome sequencing, *S. miltiorrhiza* has been considered an ideal model species for genomic and genetic studies of medicinal plants. Hydrophilic phenolic acids and lipophilic diterpenoids are the main active pharmaceutical compounds of *S. miltiorrhiza*. Hydrophilic phenolic acids such as salvianolic acid A, salvianolic acid B, and rosmarinic acid are derived from the phenylpropanoid pathway and the tyrosine-derived pathway [2,3]. Lipophilic diterpenoids such as tanshinone I, tanshinone IIA, tanshinone IIB, dihydrotanshnone I, and cryptotanshinone are diterpene quinines derived from the 2-C-methyl-d-erythritol 4-phosphate (MEP) pathway and/or the mevalonate (MVA) [4,5]. Due to great economic and medicinal value, the biosynthesis of bioactive components and its regulation mechanism in *S. miltiorrhiza* have attracted widespread interest [2,3,4,5,6,7,8,9,10,11,12]. Many genes related to the biosynthetic pathways of tanshinones and phenolic acids have been identified [4,5,13] (see Figure 1). Understanding the regulatory mechanism of phenolic acid and tanshinone biosynthesis is important for *S. miltiorrhiza* quality improvement.

The pentatricopeptide repeat (PPR) gene family exists widely in most eukaryotes including plants, mammals, and protists [14]. The number of *PPR* genes in *Trypanosoma brucei*, yeast, drosophila, and human is relatively small. It is 28, 5, 2, and 6, respectively. *Physcomitrella* has over 100 *PPRs* [15]. However, *PPR* gene numbers in plants are greatly expanded. Terrestrial plants such as *Arabidopsis*, rice, maize, and foxtail millet have 450, 477, 521, and 486 genes, respectively [16,17,18,19]. Aquatic plant duckweed has 556 genes [20]. Poplar has up to 626 genes [21]. They play a broad and essential role in posttranscriptional processes within organelles including plant growth, development, and stress responses [22]. For instance, Rf1a/b, RF5/6, and Rfn are involved in cytoplasmic male sterility and fertility restoration in rice and *Brassica napus* [23,24,25,26]. PPR8522 and GRP23 are necessary for embryogenesis in *Arabidopsis* and maize [27,28]. EMP4, Dek36, and PPR78 are required for seed development in maize [29,30,31]. WSL4 is involved in early leaf development in rice [32]. OsPPR676 is required for both pollen development and plant growth in rice [33]. PPR40, PPR96, PGN, and SOAR1 play significant roles in response to biotic and abiotic stresses in *Arabidopsis* [34,35,36,37]. MEF9, EMP5, CRR4, and PPR2263 are required for posttranscriptional processes of RNA in organelles of *Arabidopsis* and maize cells [38,39,40,41]. In addition, LOI1 regulates cytosolic and plastidial isoprenoid biosynthesis in *Arabidopsis* [42,43]. Although the physiological significance of some *PPRs* has been shown, no information is available for *PPRs* in *S. miltiorrhiza*.

In this study, 562 *SmPPRs* were genome-wide identified and characterized in *S. miltiorrhiza*. Different expression patterns were investigated. The majority of elicitor-responsive *SmPPRs* and subsets of *SmPPRs* co-expressed with phenolic acid and tanshinone biosynthetic genes were shown. Our results provide some references to further functional studies of this family gene in *S. miltiorrhiza*.

## 2. Results

### 2.1. Genome-Wide Identification and Bioinformatic Analysis of 562 SmPPRs

Through BLAST analysis of *Arabidopsis thaliana* PPRs against the current assembly of the *S. miltiorrhiza* genome and subsequent gene prediction of the retrieved genomic DNA sequences, a total of 562 *SmPPR* genes were identified. They were named *SmPPR1*–*SmPPR562*, respectively (Supplementary Table S1). These sequence data have been submitted to the GenBank databases under an accession number MH004461–MH005022. The number of *PPR* genes in *S. miltiorrhiza* is comparable with that in other plants such as *Arabidopsis*, rice, maize, and foxtail millet [16,17,18,19]. It indicates that the identified *SmPPRs* represent an almost complete set of *PPRs* in *S. miltiorrhiza* even though it may be not a fully complete set. The number of introns in *SmPPR* coding regions varied from 0 to 9 with about 88% (495/562) containing no intron, about 10% (57/562) containing one intron, and only 2% (10/562) more than one (see Supplementary Table S1). It was accorded with the previous studies showing that a noticeable feature of *PPR* genes is that most of them do not contain introns within the coding sequence [16,17,18,19,20,21]. The deduced SmPPR proteins have amino acid numbers widely ranging from 243 to 1486, theoretical *p*I values ranging between 4.47 and 9.69, and predicted molecular weights ranging from 29.98 kDa to 168.15 kDa (see Supplementary Table S1), which suggests the divergence of *SmPPRs*.

**Figure 2:**
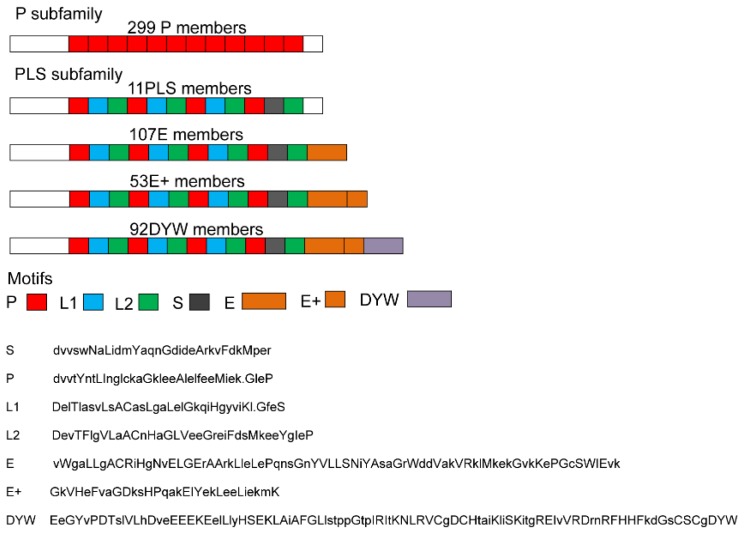
The number and structure of *S. miltiorrhiza* PPR proteins in each subfamily. Typical motifs of PPR proteins from each subfamily and subgroup are shown.

Generally, PPR proteins have 2–26 tandem arrays of a degenerate 35 amino acid repeat motif and are split into two major subfamilies including P and PLS based on the feature of motifs. P subfamily proteins have the typical P motif while PLS subfamily proteins contain the P motif and P motif-derived variants (the short (S) and the long (L) motifs). Furthermore, based on the C-terminal motifs, the PLS subfamily could be further divided into the P-L-S, E, E^+^, and DYW subgroups [16]. Motif analysis using the HMMER3.0 package showed that 299 of the 562 identified SmPPRs belonged to the P subfamily while the other 263 were members of the PLS subfamily. Among the 263 PLS SmPPRs, 11 were members of the P-L-S subgroup, 107 belonged to the E subgroup, 53 were included in the E^+^ subgroup, and the other 92 were members of the DYW subgroup (see Figure 2). We failed to construct a phylogenetic tree via the neighbor-joining method, which was consistent with previous studies that found an attempt to relate *PPR* genes with a phylogenetic tree was not meaningful since the PLS has multiple motifs that are homologous within and among them and the remaining sequences are too divergent to be aligned correctly [20].

The subcellular localization prediction showed that SmPPRs were widely located in chloroplast, mitochondrion, secretory pathway, and other locations (see Supplementary Table S1) with the majority (410/562) to be localized in the mitochondrion or chloroplast.

### 2.2. Expression Patterns of SmPPRs in Different Tissues

**Figure 3:**
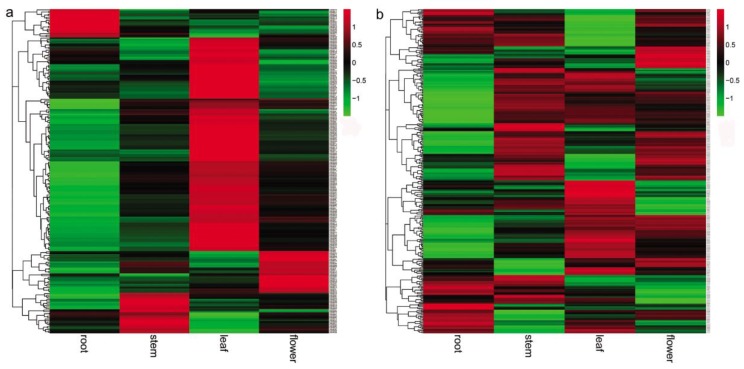
Expression patterns of *SmPPRs* in roots, stems, leaves, and flowers of *S. miltiorrhiza*. (**a**) *SmPPRs* showed tissue-specific expression. (**b**) *SmPPRs* highly expressed in at least two tissues.

It has been shown that many PPR proteins are involved in plant developmental processes [23,24,25,26,27,28,29,30,31,32,33]. In order to preliminarily know the role of *SmPPRs* in *S. miltiorrhiza* growth and development, the expression of *SmPPRs* in roots, stems, leaves, and flowers of *S. miltiorrhiza* was analyzed transcriptome-wide of which 546 were expressed (see Supplementary Table S2). Among the 546 expressed *SmPPRs*, 217 exhibited tissue-specific expressions (see Figure 3a). These include 142 expressed mainly in leaves, 29 in flowers, 27 in stems, and 19 in roots. Among these tissue-specific expression *SmPPRs*, six belong to PLS subgroup, 16 belong to E^+^ subgroup, 44 belong to DYW subgroup, 46 belong to E subgroup, and the remaining 105 belong to P subgroup (see Supplementary Table S1). These genes probably play tissue-specific roles. The other 329 were highly expressed in at least two tissues (see Figure 3b) of which five belong to the PLS subgroup, 37 belong to the E^+^ subgroup, 47 belong to the DYW subgroup, 60 belong to the E subgroup, and the remaining 180 belong to the P subgroup (see Supplementary Table S1). It indicates that they have more ubiquitous roles in *S. miltiorrhiza*. A total of 16 *SmPPRs* had RPKM (reads per kilobase of exon model per million mapped reads) values less than one in all tissues analyzed. They are pseudogenes or expressed only at specific developmental stages or under special conditions [19].

**Figure 4:**
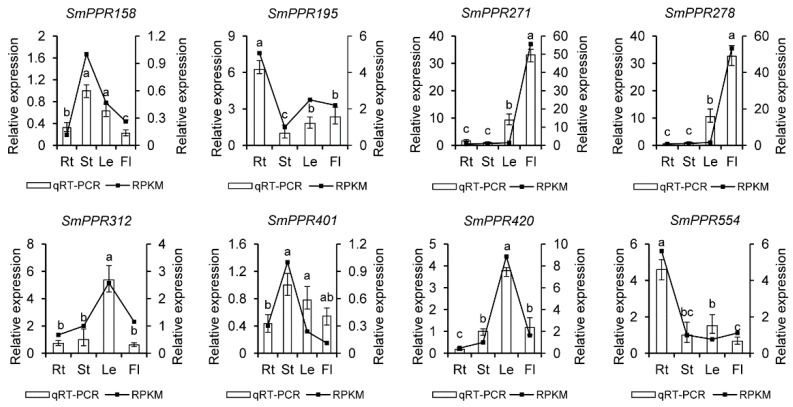
qRT-PCR analysis of eight *SmPPRs* in different tissues of *S. miltiorrhiza*. The bars represent standard errors. Different lower case letters indicate a significant difference among tissues (*p* < 0.05). Rt, root; St, stem; Le, leaf; Fl, flower.

To confirm the results from RNA-seq data, eight genes were selected for qRT-PCR analysis. It includes *SmPPR195* and *SmPPR554* mainly expressed in roots, *SmPPR158* and *SmPPR401* mainly expressed in stems, *SmPPR312* and *SmPPR420* mainly expressed in leaves, and *SmPPR271* and *SmPPR278* mainly expressed in flowers (see Figure 4). The results showed that these genes had similar trends between the results from qRT-PCR analysis and RNA-seq data, which validates the RNA-sequence results.

### 2.3. Expression of SmPPRs in Response to Salicylic Acid, Yeast Extract, and Ag^+^ Treatments

**Figure 5:**
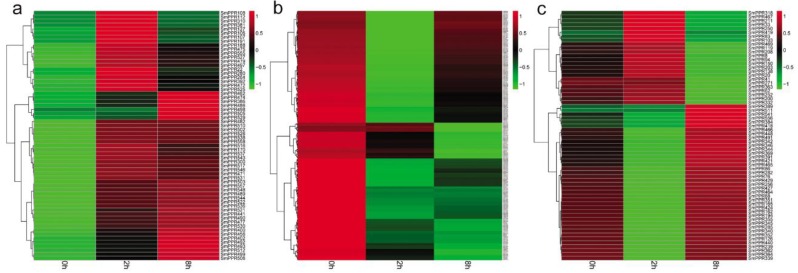
Differentially expressed *SmPPRs* in response to salicylic acid treatment. (**a**) Up-regulated genes. (**b**) Down-regulated genes. (**c**) Genes exhibited fluctuated patterns.

*PPR* genes play significant roles in plant response to stress [34,35,36,37]. However, no information is available for *SmPPRs*. In this study, to investigate the roles of *SmPPRs*, we mapped RNA-sequence data of *S. miltiorrhiza* suspension cells treated with or without salicylic acid for 0 h, 2 h, and 8 h to *SmPPRs* using the SOAP 2.0 software. A total of 558 *SmPPRs* were found to be expressed (see Supplementary Table S3). Compared with the levels in non-treated control, 349 were differentially expressed at least at one time point (see Supplementary Table S3) of which 186 belong to the P subfamily and the other 163 belong to the PLS subfamily (see Supplementary Table S1). Among them, 62 were up-regulated and 223 were down-regulated with the regulation significant in at least a time-point (see Figure 5a,b). The other 64 were up-regulated at a time-point and down-regulated at the other (see Figure 5c). The results suggest that over 62% (349/562) of *SmPPRs* are salicylic acid-responsive of which the majority were down-regulated after treatment.

**Figure 6:**
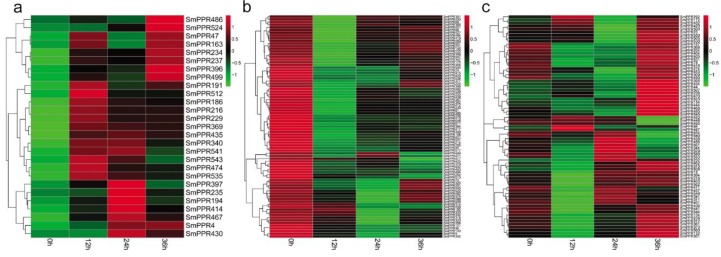
Differentially expressed *SmPPRs* in response to yeast extract and Ag^+^ treatments. (**a**) Up-regulated genes. (**b**) Down-regulated genes. (**c**) Genes exhibited fluctuated patterns.

Similarly, we mapped RNA-sequence data of *S. miltiorrhiza* hairy roots treated with or without yeast extract and Ag^+^ to *SmPPRs* for 0 h, 12 h, 24 h, and 36 h using the SOAP 2.0 software. A total of 241 *SmPPRs* had RPKM value greater than one in at least a time-point (see Supplementary Table S4). Compared to the levels in non-treated control, 183 *SmPPRs* were differentially expressed at least at one time point (see Supplementary Table S4) of which 131 belong to the P subfamily and the other 52 belong to the PLS subfamily (see Supplementary Table S1). Among them, 27 were up-regulated with the regulation to be statistically significant at least at one-time point, 83 were down-regulated, and 73 were fluctuated (see Figure 6a–c). The results suggest that over 32% (183/562) of *SmPPRs* are developed with yeast extract and Ag^+^-responsive.

Among the 349 salicylic acid-responsive and 183 yeast extract and Ag^+^-responsive *SmPPRs*, 121 responded to both salicylic acid treatment and yeast extract and Ag^+^ treatment, 228 only responded to salicylic acid, and 62 only responded to yeast extract and Ag^+^. It brings the total number of elicitor-responsive *SmPPRs* to 411 (see Figure 5 and Figure 6; Supplementary Tables S3 and S4). In poplar, on the basis of genome-wide transcriptomic analysis, 154 of the *PtrPPR* genes were induced by biotic and abiotic treatments of which 11 were chosen for verification by qRT-PCR [21]. This suggested that transcriptomic analysis was feasible. In addition, 14 of the 16 *SmPPRs* with RPKM less than one in roots, stems, leaves, and flowers of normal plants were expressed in suspension cells treated with salicylic acid and/or hairy roots treated with yeast extract and Ag^+^. It suggests that these genes are only expressed at specific developmental stages or under special conditions.

### 2.4. Co-Expression Pattern Analysis of SmPPRs with Phenolic Acid or Tanshinone Biosynthetic Genes

**Figure 7:**
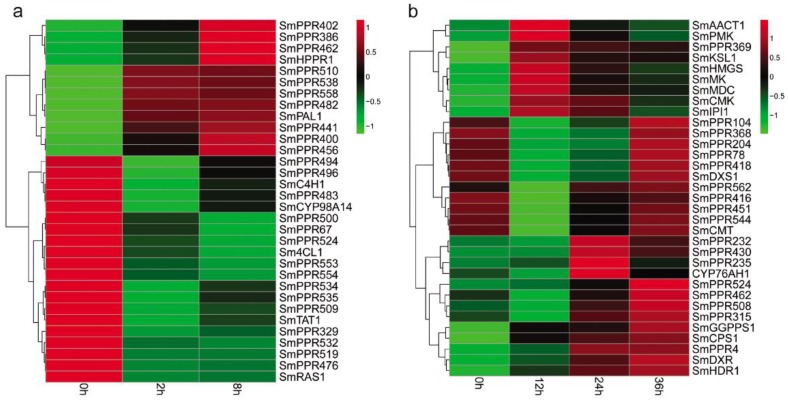
Co-expression patterns of *SmPPRs* and genes involved in phenolic acid and tanshinone biosynthesis in *S. miltiorrhiza*. (**a**) Co-expression patterns of *SmPPRs* and phenolic acid biosynthetic enzyme genes. (**b**) Co-expression patterns of *SmPPRs* and tanshinone biosynthetic enzyme genes.

Phenolic acids are a class of bioactive compounds in *S. miltiorrhiza*. Previous studies have shown that salicylic acid affects the expression of genes involved in phenolic acid biosynthesis and leads to the accumulation of these compounds in *S. miltiorrhiza* [7,9]. In this study, we found that more than 62% of the identified 562 *SmPPRs* responded to salicylic acid treatment. In order to gain insight into the relationship between *SmPPRs* and phenolic acid biosynthetic genes, we investigated the co-expression patterns of 349 salicylic acid-responsive *SmPPRs* and phenolic acid biosynthetic genes including *SmPAL1*, *SmC4H1*, *Sm4CL1*, *SmTAT1*, *SmHPPR1*, *SmRAS1*, and *SmCYP98A14* [13]. As a result, 25 co-expressed *SmPPRs* were identified (see Figure 7a).

Tanshinones are the other class of bioactive substances in *S. miltiorrhiza*. Many tanshinone biosynthetic enzyme genes responded to yeast extract and Ag^+^ treatment [6,44]. In this study, we identified 183 yeast extract and Ag^+^-responsive *SmPPRs*. The relationship between *SmPPRs* and tanshinone biosynthetic genes were investigated. It results in the identification of 18 co-expressed *SmPPRs* [13] (see Figure 7b).

Co-expression pattern analysis showed that 23 *SmPPRs* were co-expressed with phenolic acid biosynthetic enzyme genes only, 16 *SmPPRs* were co-expressed with tanshinone biosynthetic enzyme genes only, and two *SmPPRs* were co-expressed with both phenolic acid and tanshinone biosynthetic enzyme genes (see Figure 7). 

## 3. Discussion

In this study, 562 *SmPPRs* were identified genome-widely in *S. miltiorrhiza* of which 299 members belong to the P subfamily and the others were members of the PLS subfamily. Among the 263 members of the PLS subfamily, 11 were members of the P-L-S subgroup, 107 belonged to the E subgroup, 53 were included in the E^+^ subgroup, and the other 92 were members of the DYW subgroup (see Figure 2, Supplementary Table S1). However, in *Arabidopsis*, 251 members are from the P subfamily and 199 members are from the PLS subfamily including six members of the P-L-S subgroup, 47 members of the E subgroup, 59 members of the E^+^ subgroup, and 87 members of the DYW subgroup [16]. Comparative analysis of PPR numbers in each subfamily showed that *S. miltiorrhiza* had more P, P-L-S, and E PPRs than *Arabidopsis* and the number of P-L-S SmPPRs was about twice that of P-L-S AtPPRs and the number of E SmPPRs was more than twice that of P-L-S AtPPRs, which suggests the member of these subfamilies expanded in *S. miltiorrhiza*.

It has been reported that some *PPRs* play important roles in plant developmental processes including cytoplasmic male sterility and fertility restoration, embryogenesis, and seed development [45]. In this study, the tissue-specific expression patterns of *SmPPRs* in roots, stems, leaves, and flowers in *S. miltiorrhiza* were investigated based on transcriptome data, which shows that the majority of *SmPPRs* were expressed ubiquitously with the exception of a few genes expressed in specific tissues (see Figure 3, Supplementary Table S2). It is in line with previous studies in maize [19], which suggests that *SmPPRs* are multifunctional and are involved in a wide range of biological processes.

More research has shown that *PPRs* are involved in plant responses to stresses and secondary metabolism [34,35,36,37,42,43]. However, the function of many *PPRs* is still unknown and there is a lack of knowledge about *SmPPRs*. Previous studies suggested that salicylic acid could inhibit the activity of plasma membrane H^+^-ATPase, could make cell generate oxidative stress, could promote the synthesis of phenolic acids, and up-regulate the expression of *PAL*, *TAT*, and *RAS* in *S. miltiorrhiza* cell culture [7,9]. Ag^+^ could trigger a burst of reactive oxygen species (ROS) and yeast extract could improve the antioxidant defense of *S. miltiorrhiza* hairy roots [6,11]. The combined use of yeast extract and Ag^+^ was more effective than single yeast extract or Ag^+^ in the hairy root cultures of *S. miltiorrhiza* [44,45]. They could promote the accumulation of tanshinones in *S. miltiorrhiza* and stimulate the activities of HMGR and DXS enzymes [6]. These results suggest that salicylic acid, yeast extract, and Ag^+^ are effective elicitors for bioactive compound production and related gene expression in *S. miltiorrhiza*. In this study, 349 *SmPPRs* were responsive to salicylic acid treatment and 183 *SmPPRs* were responsive to yeast extract and Ag^+^ treatment, which indicates the importance of *SmPPRs* in *S. miltiorrhiza* stress responses and secondary metabolites. Co-expression analysis has previously been described to be a suitable tool for gene function identification [46]. Various genes such as *Arabidopsis* starch metabolism-related genes [47], rice cell-wall formation-related genes [48], and potato starch biosynthesis-associated transcription factor genes [49], were identified based on co-expression analysis. Gene co-expression analysis showed that 25 salicylic acid-responsive *SmPPRs* were co-expressed with phenolic acid biosynthetic genes and 18 yeast extract and Ag^+^-responsive *SmPPRs* were co-expressed with tanshinone biosynthetic genes (see Figure 7). It includes two co-expressed with both phenolic acid and tanshinone biosynthetic enzyme genes, which brings the total number of co-expressed *SmPPRs* to 41. These *SmPPRs* could be involved in phenolic acid and/or tanshinone biosynthesis in *S. miltiorrhiza*. Subcellular localization prediction showed these SmPPR proteins mainly located in mitochondrion and chloroplast (see Supplementary Table S1). They could be related with the respiratory cytochrome pathway or the photosynthesis of chloroplasts to regulate the biosynthesis of phenolic acid and tanshinone [42,43]. They also could be through the mitochondrial electron transport system or hormonal responses to regulate plant environmental stresses - responsive [35,36,37]. Further physiological and biochemical analysis will be performed to verify the functions of these *SmPPRs* in the future.

## 4. Materials and Methods

### 4.1. Plant Materials

*Salvia miltiorrhiza* Bunge (line 993) with whole genome sequence available was grown in a field nursery at the Institute of Medicinal Plant Development (Beijing, China). Roots, stems, leaves, and flowers were collected from two-year-old plants and stored in liquid nitrogen until use.

### 4.2. Genome-Wide Identification of SmPPRs

*Arabidopsis* PPR (AtPPR) protein sequences were downloaded from TAIR (http://www.arabidopsis.org/). BLAST analysis of AtPPR proteins against the genome database of *S. miltiorrhiza* (line 993) [50] was carried out using tBLASTn. An e-value cut-off of e^−10^ was applied. Gene models of *SmPPRs* were predicted based on the alignments between the retrieved DNA sequences and PPR proteins from other plant species via BLASTx. The predicted gene models were examined and comparatively analyzed with the genome database of the other *S. miltiorrhiza* line (http://www.herbal-genome.cn). The domain information of SmPPR proteins was analyzed using the software package HMMER 3.0 (http://hmmer.janelia.org/software/archive). The HMMbuild program in the HMMER 3.0 package was used for producing the matrices specific to each type of SmPPRs. Motifs were searched using the HMMsearch program in the HMMER 3.0 package. The gene structures were analyzed using the Gene Structure Display Server (GSDS 2.0, http://gsds.cbi.pku.edu.cn/index.php). Subcellular localization of SmPPR proteins was predicted with TargetP version 1.1 (http://www.cbs.dtu.dk/services/TargetP/). The molecular weight (MW) and theoretical isoelectric point (*p*I) were predicted via the compute *p*I/MW tool on the ExPASy server (http://web.expasy.org/compute_pi/).

### 4.3. Expression Analysis of SmPPRs

Expression patterns of *SmPPR* genes in different organs were analyzed using the transcriptome datasets of roots, stems, leaves, and flowers of *S. miltiorrhiza* downloaded from GenBank (SRP051564, SRP028388). The changes of *SmPPR* expression levels in response to stress treatments were analyzed using the transcriptome datasets of suspension cells treated with salicylic acid (SA, 0.16 mM) (SRX1423774) for 0 h, 2 h, and 8 h and hairy roots treated with yeast extract (100 µg/mL) and Ag^+^ (30 µM) (SRR924662) for 0 h, 12 h, 24 h, and 36 h. RNA-sequence reads were mapped to *SmPPRs* using SOAP 2.0 [51] and analyzed as described previously [12]. Co-expression pattern analysis of *SmPPR* genes with phenolic acid and tanshinone biosynthetic genes in *S. miltiorrhiza* was analyzed using the software package of Co-expression Pattern Clustering Analysis in the BMKCloud cloud server (http://www.biocloud.net/). The abundance of transcripts was estimated by RPKM. Genes with RPKM value greater than one in at least a tissue was considered to be expressed. To draw heat maps, the value of RPKM for each gene was normalized between −1.0 and 1.0 using the R package version 3.4.1 and analyzed for differential expression by the Fisher’s exact test. *p* < 0.05 was considered as differentially expressed.

### 4.4. Quantitative Real-Time Reverse Transcription-PCR (qRT-PCR)

Gene expression was analyzed using qRT-PCR. Total RNA was extracted from prepared samples using the Plant RNA EASYspin Plus (Aidlab biotech, Beijing, China). RNA integrity was analyzed on a 1.2% agarose gel and its quantity was determined using a NanoDrop 2000C Spectrophotometer (Thermo Scientific, Waltham, MA, USA). Reverse transcription was carried out using the PrimeScript™ RT reagent kit with gDNA Eraser (Perfect Real Time) (Takara, Dalian, China). PCR was performed using the SYBR^®^ Premix Ex Taq™ II (Tli RNaseH Plus, Shiga, Japan) (TaKaRa) as described previously [12]. Gene-specific primers for *SmPPRs* were listed in Supplementary Table S5. The length of amplicons was between 80 bp and 250 bp. *SmUBQ10* was used as an internal control. qRT-PCRs were performed in three biological replicates with three technical replicates each. The expression level in stems was set to one and the levels in other tissues were given relative to this. The relative expression levels of genes were calculated by the 2^−ΔΔCt^ method. ANOVA (analysis of variance) was calculated using SPSS (Version 19.0, IBM, Chicago, IL, USA). *p* < 0.05 was considered statistically significant.

## 5. Conclusions

In this study, bioinformatic analysis for *PPR* gene family in *S. miltiorrhiza* was performed. A total of 562 PPR protein genes were identified in *S. miltiorrhiza* genome. Gene structure and classification showed important features for this family. Tissue-specific expression analysis indicated that *SmPPRs* have more ubiquitous roles in *S. miltiorrhiza*. The expression pattern in response to different elicitors revealed *SmPPRs* might be involved in stress defense and secondary metabolites. Furthermore, some *SmPPRs* co-expressed with phenolic acid and tanshinone biosynthetic genes were shown. The results provide valuable information for future studies on characterizing the biological functions of PPR protein genes in *S. miltiorrhiza*.

## Supplementary Materials

The following are available online. Table S1: Sequence features of the *PPR* gene family members in *S. miltiorrhiza*. Table S2: The expression levels of *SmPPR* genes in different tissues (RPKM). Table S3: RPKM value for *SmPPR* expression in *S. miltiorrhiza* suspension cell treated with or without salicylic acid. Table S4: RPKM value for *SmPPR* expression in hairy roots treated with or without yeast extract and Ag^+^. Table S5: Primers used for qRT-PCR.

## References

[1] Zhang Z., Lam T.N., Zuo Z. (2013). Danshen: An overview of its chemistry, pharmacology, pharmacokinetics, and clinical use. J. Clin. Pharm..

[2] Hou X., Shao F., Ma Y., Lu S. (2013). The phenylalanine ammonia-lyase gene family in *salvia miltiorrhiza*: Genome-wide characterization, molecular cloning and expression analysis. Mol. Biol. Rep..

[3] Deng Y., Lu S. (2017). Biosynthesis and regulation of phenylpropanoids in plants. Crit. Rev. Plant Sci..

[4] Ma Y., Yuan L., Wu B., Li X., Chen S., Lu S. (2012). Genome-wide identification and characterization of novel genes involved in terpenoid biosynthesis in *Salvia miltiorrhiza*. J. Exp. Bot..

[5] Zhang L., Lu S. (2017). Overview of medicinally important diterpenoids derived from plastids. Mini-Rev. Med. Chem..

[6] Ge X., Wu J. (2005). Tanshinone production and isoprenoid pathways in *Salvia miltiorrhiza*, hairy roots induced by Ag^+^, and yeast elicitor. Plant Sci..

[7] Dong J., Wan G., Liang Z. (2010). Accumulation of salicylic acid-induced phenolic compounds and raised activities of secondary metabolic and antioxidative enzymes in *Salvia miltiorrhiza* cell culture. J. Biot..

[8] Liang Z.S., Yang D.F., Liang X., Zhang Y.J., Liu Y., Liu F.H. (2012). Roles of reactive oxygen species in methyl jasmonate and nitric oxide-induced tanshinone production in *Salvia miltiorrhiza* hairy roots. Plant Cell Rep..

[9] Li X., Guo H., Qi Y., Liu H., Zhang X., Ma P., Liang Z., Dong J. (2016). Salicylic acid-induced cytosolic acidification increases the accumulation of phenolic acids in *Salvia miltiorrhiza* cells. Plant Cell Tissue Org. Cult..

[10] Liu M., Lu S. (2016). Plastoquinone and ubiquinone in plants: Biosynthesis, physiological function and metabolic engineering. Front. Plant Sci..

[11] Wang Y., Shen Y., Shen Z., Zhao L., Ning D., Jiang C., Zhao R., Huang L. (2016). Comparative proteomic analysis of the response to silver ions and yeast extract in *Salvia miltiorrhiza* hairy root cultures. Plant Physiol. Biochem..

[12] Li C., Li D., Li J., Shao F., Lu S. (2017). Characterization of the polyphenol oxidase gene family reveals a novel microRNA involved in posttranscriptional regulation of *PPOs* in *Salvia miltiorrhiza*. Sci. Rep..

[13] Ma X.H., Ma Y., Tang J.F., He Y.L., Liu Y.C., Ma X.J., Shen Y., Cui G.H., Lin H.X., Rong Q.X. (2015). The biosynthetic pathways of tanshinones and phenolic acids in *Salvia miltiorrhiza*. Molecules.

[14] Hayes M.L., Karolyn G., Mulligan R. (2012). Molecular evolution of pentatricopeptide repeat genes reveals truncation in species lacking an editing target and structural domains under distinct selective pressures. BMC Evol. Biol..

[15] Sugita M., Ichinose M., Ide M., Sugita C. (2013). Architecture of the *PPR* gene family in the moss *Physcomitrella patens*. RNA Biol..

[16] Lurin C., Andrés C., Aubourg S., Bellaoui M., Bitton F., Bruyère C., Caboche M., Debast C., Gualberto J., Hoffmann B. (2004). Genome-wide analysis of *Arabidopsis* pentatricopeptide repeat proteins reveals their essential role in organelle biogenesis. Plant Cell.

[17] O’Toole N., Hattori M., Andres C., Iida K., Lurin C., Schmitz-Linneweber C., Sugita M., Small I. (2008). On the expansion of the pentatricopeptide repeat gene family in plants. Mol. Biol. Evol..

[18] Liu J.M., Xu Z.S., Lu P.P., Li W.W., Chen M., Guo C.H., Ma Y.Z. (2016). Genome-wide investigation and expression analyses of the pentatricopeptide repeat protein gene family in foxtail millet. BMC Genom..

[19] Wei K., Han P. (2016). Pentatricopeptide repeat proteins in maize. Mol. Breed..

[20] Wang W., Wu Y., Messing J. (2016). Genome-wide analysis of pentatricopeptide-repeat proteins of an aquatic plant. Planta.

[21] Xing H., Fu X., Yang C., Tang X., Guo L., Li C., Xu C., Luo K. (2018). Genome-wide investigation of pentatricopeptide repeat gene family in poplar and their expression analysis in response to biotic and abiotic stresses. Sci. Rep..

[22] Manna S. (2015). An overview of pentatricopeptide repeat proteins and their applications. Biochimie.

[23] Wang Z., Zou Y., Li X., Zhang Q., Chen L., Wu H., Su D., Chen Y., Guo J., Luo D. (2006). Cytoplasmic male sterility of rice with boro II cytoplasm is caused by a cytotoxic peptide and is restored by two related PPR motif genes via distinct modes of mRNA silencing. Plant Cell.

[24] Hu J., Wang K., Huang W., Liu G., Gao Y., Wang J., Huang Q., Ji Y., Qin X., Wan L. (2012). The rice pentatricopeptide repeat protein RF5 restores fertility in Hong-Lian cytoplasmic male-sterile lines via a complex with the glycine-rich protein GRP162. Plant Cell.

[25] Huang W., Yu C., Hu J., Wang L., Dan Z., Zhou W., He C., Zeng Y., Yao G., Qi J. (2015). Pentatricopeptide-repeat family protein RF6 functions with hexokinase 6 to rescue rice cytoplasmic male sterility. Proc. Natl. Acad. Sci. USA.

[26] Liu Z., Dong F., Wang X., Wang T., Su R., Hong D., Yang G. (2017). A pentatricopeptide repeat protein restores nap cytoplasmic male sterility in *Brassica napus*. J. Exp. Bot..

[27] Ding Y.H., Liu N.Y., Tang Z.S., Liu J., Yang W.C. (2006). *Arabidopsis* GLUTAMINE-RICH PROTEIN23 is essential for early embryogenesis and encodes a novel nuclear PPR motif protein that interacts with RNA polymerase II dubunit III. Plant Cell.

[28] Sosso D., Canut M., Gendrot G., Dedieu A., Chambrier P., Barkan A., Consonni G., Rogowsky P.M. (2012). PPR8522 encodes a chloroplast-targeted pentatricopeptide repeat protein necessary for maize embryogenesis and vegetative development. J. Exp. Bot..

[29] Gutiérrez-Marcos J.F., Dal P.M., Giulini A., Costa L.M., Gavazzi G., Cordelier S., Sellam O., Tatout C., Paul W., Perez P. (2007). Empty pericarp4 encodes a mitochondrion-targeted pentatricopeptide repeat protein necessary for seed development and plant growth in maize. Plant Cell.

[30] Wang G., Zhong M., Shuai B., Song J., Zhang J., Han L., Ling H., Tang Y., Wang G., Song R. (2017). E+ subgroup PPR protein defective kernel 36 is required for multiple mitochondrial transcripts editing and seed development in maize and *Arabidopsis*. New Phytol..

[31] Zhang Y.F., Suzuki M., Sun F., Tan B.C. (2017). The mitochondrion-targeted PENTATRICOPEPTIDE REPEAT78 protein is required for nad5 mature mRNA stability and seed development in maize. Mol. Plant.

[32] Wang Y., Ren Y.L., Zhou K.N., Liu L.L., Wang J.L., Xu Y., Zhang H., Zhang L., Feng Z.M., Wang L.W. (2017). WHITE STRIPE LEAF4 encodes a novel P-type PPR protein required for chloroplast biogenesis during early leaf development. Front. Plant Sci..

[33] Liu Y.J., Liu X., Chen H., Zheng P., Wang W., Wang L., Zhang J., Tu J. (2017). A plastid-localized pentatricopeptide repeat protein is required for both pollen development and plant growth in rice. Sci. Rep..

[34] Zsigmond L., Rigó G., Szarka A., Székely G., Otvös K., Darula Z., Medzihradszky K.F., Koncz C., Koncz Z., Szabados L. (2008). *Arabidopsis* PPR40 connects abiotic stress responses to mitochondrial electron transport. Plant Physiol..

[35] Laluk K., Abuqamar S., Mengiste T. (2011). The *Arabidopsis* mitochondria-localized pentatricopeptide repeat protein PGN functions in defense against necrotrophic fungi and abiotic stress tolerance. Plant Physiol..

[36] Jiang S.C., Mei C., Liang S., Yu Y.T., Lu K., Wu Z., Wang X.F., Zhang D.P. (2015). Crucial roles of the pentatricopeptide repeat protein SOAR1 in *Arabidopsis* response to drought, salt and cold stresses. Plant Mol. Biol..

[37] Liu J.M., Zhao J.Y., Lu P.P., Chen M., Guo C.H., Xu Z.S., Ma Y.Z. (2016). The E-subgroup pentatricopeptide repeat protein family in *Arabidopsis thaliana* and confirmation of the responsiveness PPR96 to abiotic stresses. Front. Plant Sci..

[38] Kotera E., Tasaka M., Shikanai T. (2005). A pentatricopeptide repeat protein is essential for RNA editing in chloroplasts. Nature.

[39] Takenaka M. (2010). MEF9, an E-subclass pentatricopeptide repeat protein, is required for an RNA editing event in the nad7 transcript in mitochondria of *Arabidopsis*. Plant Physiol..

[40] Sosso D., Mbelo S., Vernoud V., Gendrot G., Dedieu A., Chambrier P., Dauzat M., Heurtevin L., Guyon V., Takenaka M. (2012). PPR2263, a DYW-subgroup pentatricopeptide repeat protein, is required for mitochondrial nad5 and cob transcript editing, mitochondrion biogenesis, and maize growth. Plant Cell.

[41] Liu Y.J., Xiu Z.H., Meeley R., Tan B.C. (2013). Empty pericarp5 encodes a pentatricopeptide repeat protein that is required for mitochondrial RNA editing and seed development in maize. Plant Cell.

[42] Kobayashi K., Suzuki M., Tang J., Nagata N., Ohyama K., Seki H., Kiuchi R., Kaneko Y., Nakazawa M., Matsui M. (2007). Lovastatin insensitive 1, a novel pentatricopeptide repeat protein, is a potential regulatory factor of isoprenoid biosynthesis in *Arabidopsis*. Plant Cell Physiol..

[43] Tang J., Kobayashi K., Suzuki M., Matsumoto S., Muranaka T. (2010). The mitochondrial PPR protein LOVASTATIN INSENSITIVE 1 plays regulatory roles in cytosolic and plastidial isoprenoid biosynthesis through RNA editing. Plant J..

[44] Cheng Q., He Y., Li G., Liu Y., Gao W., Huang L. (2013). Effects of combined elicitors on tanshinone metabolic profiling and *SmCPS* expression in *Salvia miltiorrhiza* hairy root cultures. Molecules.

[45] Kai G., Xu H., Wang J., Zhou C., Zhou W., Qi Y., Xiao J., Wang Y., Zhang L. (2012). Molecular mechanism of elicitor-induced tanshinone accumulation in *Salvia miltiorrhiza* hairy root cultures. Acta Physiol. Plant..

[46] Usadel B., Obayashi T., Mutwil M., Giorgi F.M., Bassel G.W., Tanimoto M., Chow A., Steinhauser D., Persson S., Provart N.J. (2009). Co-expression tools for plant biology: Opportunities for hypothesis generation and caveats. Plant Cell Environ..

[47] Li L., Wurtele E.S. (2007). Genome wide co-expression among the starch debranching enzyme genes *AtISA1*, *AtISA2*, and *AtISA3* in *Arabidopsis thaliana*. J. Exp. Bot..

[48] Chandran A.K.N., Jeong H.Y., Jung K.H., Lee C. (2016). Development of functional modules based on co-expression patterns for cell-wall biosynthesis related genes in rice. J. Plant Biol..

[49] Harsselaar J.K.V., Lorenz J., Senning M., Sonnewald U., Sonnewald S. (2017). Genome-wide analysis of starch metabolism genes in potato (*Solanum tuberosum* L.). BMC Genom..

[50] Xu H., Song J., Luo H., Zhang Y., Li Q., Zhu Y., Xu J., Li Y., Song C., Wang B. (2016). Analysis of the genome sequence of the medicinal plant *Salvia miltiorrhiza*. Mol. Plant.

[51] Li R., Yu C., Li Y., Lam T.W., Yiu S.M., Kristiansen K., Wang J. (2009). SOAP2: An improved ultrafast tool for short read alignment. Bioinformatics.

